# Functionalized PHB granules provide the basis for the efficient side-chain cleavage of cholesterol and analogs in recombinant *Bacillus megaterium*

**DOI:** 10.1186/s12934-015-0300-y

**Published:** 2015-07-29

**Authors:** Adrian Gerber, Michael Kleser, Rebekka Biedendieck, Rita Bernhardt, Frank Hannemann

**Affiliations:** Centre for Human and Molecular Biology, Institute of Biochemistry, Saarland University, 66123 Saarbrücken, Germany; Institute of Microbiology, Technische Universität Braunschweig, 38106 Braunschweig, Germany

**Keywords:** Cholesterol, Cytochrome P450, *Bacillus megaterium*, Pregnenolone, PHB

## Abstract

**Background:**

Cholesterol, the precursor of all steroid hormones, is the most abundant steroid in vertebrates and exhibits highly hydrophobic properties, rendering it a difficult substrate for aqueous microbial biotransformations. In the present study, we developed a *Bacillus megaterium* based whole-cell system that allows the side-chain cleavage of this sterol and investigated the underlying physiological basis of the biocatalysis.

**Results:**

CYP11A1, the side-chain cleaving cytochrome P450, was recombinantly expressed in the Gram-positive soil bacterium *B. megaterium* combined with the required electron transfer proteins. By applying a mixture of 2-hydroxypropyl-β-cyclodextrin and *Quillaja* saponin as solubilizing agents, the zoosterols cholesterol and 7-dehydrocholesterol, as well as the phytosterol β-sitosterol could be efficiently converted to pregnenolone or 7-dehydropregnenolone. Fluorescence-microscopic analysis revealed that cholesterol accumulates in the carbon and energy storage-serving poly(3-hydroxybutyrate) (PHB) bodies and that the membrane proteins CYP11A1 and its redox partner adrenodoxin reductase (AdR) are likewise localized to their surrounding phospholipid/protein monolayer. The capacity to store cholesterol was absent in a mutant strain devoid of the PHB-producing polymerase subunit PhaC, resulting in a drastically decreased cholesterol conversion rate, while no effect on the expression of the recombinant proteins could be observed.

**Conclusion:**

We established a whole-cell system based on *B. megaterium*, which enables the conversion of the steroid hormone precursor cholesterol to pregnenolone in substantial quantities. We demonstrate that the microorganism’s PHB granules, aggregates of bioplastic coated with a protein/phospholipid monolayer, are crucial for the high conversion rate by serving as substrate storage. This microbial system opens the way for an industrial conversion of the abundantly available cholesterol to any type of steroid hormones, which represent one of the biggest groups of drugs for the treatment of a wide variety of diseases.

**Electronic supplementary material:**

The online version of this article (doi:10.1186/s12934-015-0300-y) contains supplementary material, which is available to authorized users.

## Background

The chemical cleavage of a carbon–carbon bond is extremely difficult to execute, due to the inertness of these highly thermostable bonds and the constraint spatial properties of its σ-orbitals [1]. There are, however, several enzymes that can specifically catalyze this reaction type, a prime example being the cytochrome P450 11A1, which is able to cleave the non-activated side-chain of cholesterol [2], leading to pregnenolone, the central compound in the biogenesis of steroid hormones in all vertebrates. Its derivatives build a group of about 300 approved steroid drugs which are commonly used for the treatment of a variety of diseases since the 1950’s [3]. For example, glucocorticoids are administered for the treatment of rheumatoid arthritis, asthma, eczema, anaphylactic shock, and Addison’s disease; steroidal sex hormones for the treatment of male sexual organ dysfunction, gynecopathy and for contraception; and anabolic steroid hormones for the treatment of diuresis, sinusitis and the improvement of protein metabolism.

Owing to the great demand for steroidal drugs, the production developed steadily since Merck first introduced cortisone as a drug [4]. Today, the steroid production represents one of the largest sectors of medical products manufactured by the pharmaceutical industry and is one of the best examples of the successful combination of chemical and microbial technology in large scale industrial processes. Although some of the applied microbial conversions are well-established, efforts are ongoing either to improve the existing bioconversions or to develop new economically interesting processes such as the microbial side-chain cleavage of natural sterols to produce pregnenolone. The application of low-cost extracts of the most abundant sterol cholesterol or its plant-derived analogs as substrates has been hindered so far by the hydrophobic nature of these substances and, subsequently, their low uptake rate into microbial cells. Hence, to the best our knowledge, so far no recombinant whole-cell system has been described, which can efficiently convert these compounds to pregnenolone. Attempts to utilize recombinant *E. coli* for pregnenolone production yielded only product concentrations in the µg/L range after 24 h [5]. In recent years, efforts have been made to produce steroid hormones in *Saccharomyces cerevisiae* by engineering the sterol biosynthesis pathway of the organism itself and thus endogenously providing the substrates for the recombinant enzymes [6–8]. However, this leads to the accumulation of unwanted side-products like ergosterol and brassicasterol that cannot be converted to pregnenolone [9] by CYP11A1, due to the presence of a double bond between carbon atoms 20 and 22 in the side-chain of these substrates, resulting in a reduced efficiency of the whole-cell system.

The selected host in this work, *Bacillus megaterium*, is an aerobic, Gram-positive bacterium, which is mostly found in soil. In the past two decades this non-pathogenic microorganism has gained attention as a host for biotechnological applications owing to its high capacity for recombinant protein production, high plasmid stability and broad range of substrates [10]. Additionally, its large cell size with a 100-fold higher volume compared to *Escherichia coli* cells allows detailed microscopic studies [11]. We constructed a *B. megaterium* strain that recombinantly produces bovine CYP11A1 and its redox partners adrenodoxin (Adx) and adrenodoxin reductase (AdR) [12].

CYP11A1 belongs to the large and diverse superfamily of P450 enzymes that act as external monooxygenases and catalyze a broad variety of reactions. They activate molecular oxygen through their heme iron and catalyze the oxidation of organic substances during biotransformation of xenobiotics, metabolic activation of carcinogens and biosynthesis of steroids [13, 14]. CYP11A1, as a mitochondrial P450 enzyme, receives the necessary electrons for catalysis from NADPH via a typical class I redox system [15], in which the [2Fe2S] ferredoxin Adx transfers electrons from an NADPH-dependent ferredoxin reductase, AdR, to the heme iron in CYP11A1. The terminal electron acceptor CYP11A1 takes up the water-insoluble cholesterol at the inner mitochondrial membrane and converts it to the less hydrophobic product pregnenolone, removing the unpolar side chain by cleaving the 20,22 bond of the steroid. This reaction is mainly carried out in the male and female reproductive tissues and the adrenal gland and represents the first, rate-limiting and hormonally regulated step in the synthesis of all steroid hormones in mammals. Pregnenolone is the precursor of all glucocorticoids, mineralocorticoids, and steroidal sex hormones. In addition, pregnenolone serves as a neurosteroid, involved in memory and neurological recovery by promoting microtubule polymerization and cell migration [16]. CYP11A1 is also expressed in the human skin, forming a metabolically active barrier by activating or inactivating biologically relevant molecules. The enzyme transforms 7-dehydrocholesterol (7DHC), the precursor of vitamin D3, to 7-dehydropregnenolone (7DHP), whose photo-transformed 5,7-diene derivatives exhibit an anti-proliferative effect against melanoma and leukemia cells [17].

The efficient substrate conversion presented in this work is based on the presence of granules in the cytosol of *B. megaterium* [18–20], predominantly containing poly(3-hydroxybutyrate) (PHB), a form of polyhydroxyalkanoic acid (PHA). These natural water-insoluble inclusions are formed by various bacteria and serve as carbon and energy storage during times of oversupply with carbon sources. They are complex subcellular organelles consisting of a PHB core surrounded by a monolayer of phospholipids and essential proteins for PHA metabolism [21]. These proteins are responsible for the biosynthesis or the degradation of PHA and include subunits of the PHA synthase, phasins, PHA depolymerizing enzymes, and regulatory proteins [22, 23]. Among them, PHA synthase is the key enzyme of PHA synthesis. It accepts coenzyme A thioesters of hydroxyalkanoic acids (HA) as substrates and catalyzes the polymerization of HAs into PHA while simultaneously releasing CoA. PHA synthases currently are divided into four classes depending on their subunit composition and substrate specificity, whereas *Bacillus* sp. express dimeric class IV PHA synthases consisting of the subunits PhaC and PhaR, respectively.

Due to their interesting physical and material properties the polyesters derived from PHB granules are considered for several applications in medicine, pharmacy, agriculture as well as the packaging and food industry [24]. The granules have also attracted interest for the production of biocompatible and biodegradable nanoparticles, which can be applied for drug delivery, diagnostics, bioseparation and protein immobilization [25].

In the present paper, we are demonstrating the efficient in vivo conversion of cholesterol or analogs by a recombinant microorganism, utilizing the bacterium’s PHB granules for substrate storage and embedding of the heterologous membrane proteins, without modifying these proteins with additional affinity tags. This biocatalyst system allows the production of the steroid precursor pregnenolone starting from low-cost crude extracts of cholesterol or plant-derived sterols like β-sitosterol or campesterol. After this crucial step, pregnenolone can further be transformed to a variety of desired end-products either by chemical means or by the application of further steroid converting enzymes.

## Results

### Establishment of a whole-cell system for the conversion of cholesterol and analogs using *Bacillus megaterium*

**Fig. 1:**
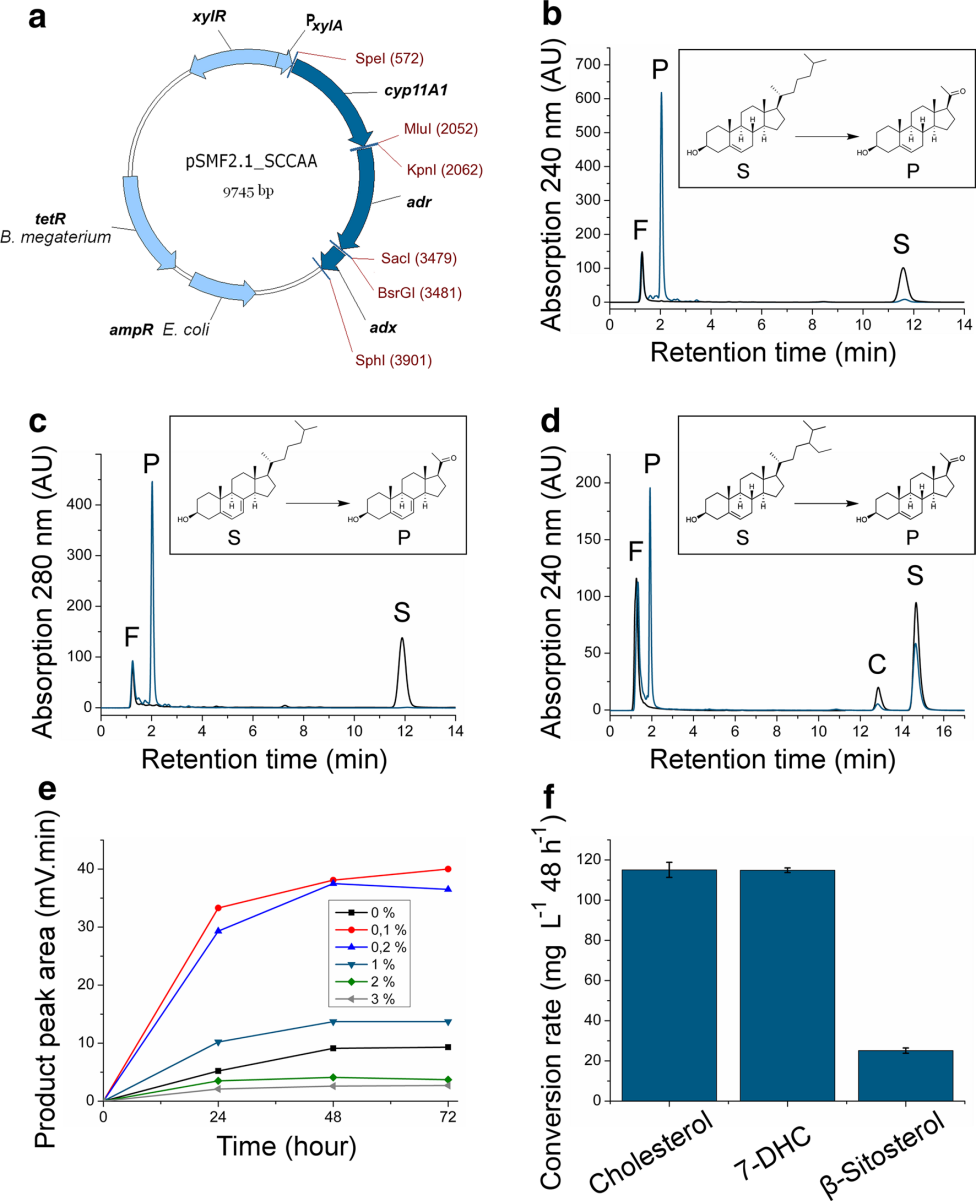
Characterization of *B. megaterium* (strain MS941) whole-cell system for the conversion of cholesterol and analogs. **a** Tricistronic vector pSMF2.1_SCCAA encoding side-chain cleavage enzyme CYP11A1 as well as redox partners AdR and Adx under the control of a xylose-inducible promoter (*P*_*xylA*_, promoter xylose utilization operon; *xylR*, xylose repressor; *tetR*, tetracycline resistance gene; *ampR*, ampicillin resistance gene). **b** HPLC-chromatograms of the conversion of cholesterol after 48 h (*light gray* strain MS941 transformed with pSMF2.1_SCCAA, *black* untransformed strain, *S* cholesterol, *P* pregnenolone, *F* flow-through peak). **c** HPLC-chromatograms of the conversion of 7-DHC after 48 h (*light gray* strain MS941 transformed with pSMF2.1_SCCAA, *black* untransformed strain, *S* 7-DHC, *P* 7-DHP, *F* flow-through peak). **d** HPLC chromatograms of the conversion of β-sitosterol after 48 h (*light gray* strain MS941 transformed with pSMF2.1_SCCAA, *black* untransformed strain, *S* β-sitosterol, *P* pregnenolone, *C* campesterol contained in the sample as minor impurity, *F* flow-through peak). **e** Effect of different final concentrations of *Quillaja* saponin (*inset*) on product yield. The substrate was dissolved in a 45% 2-hydroxypropyl-β-cyclodextrin with varying saponin concentrations and added to the cultures directly after protein induction. Pregnenolone yield after cholesterol conversion was determined by HPLC. **f** Conversion rates of substrates cholesterol, 7-DHC and β-sitosterol after 48 h. *Error bars* indicate standard deviation of triplicate biological experiments.

In order to provide *B*. *megaterium* with the ability to perform the side-chain cleavage reaction on steroids, we transformed protoplasts of the strain MS941 (DSMZ: German Collection of Microorganisms and Cell Cultures) with the tricistronic vector pSMF2.1_SCCAA. This plasmid contains an operon consisting of genes for bovine CYP11A1 as well as its natural redox partners Adx and AdR under the control of a xylose-inducible promoter (Fig. 1a). Three model substrates were chosen for a potential whole-cell conversion with this recombinant strain: the zoosterols cholesterol and 7-dehydrocholesterol (7-DHC) as well as the phytosterol β-sitosterol (Fig. 1b–d, insets). All steroids were dissolved in an aqueous solution of 2-hydroxypropyl-β-cyclodextrin and *Quillaja* saponin. Simultaneous to protein induction, the substrate solution was added to the shake-flask cultivated recombinant *B. megaterium* strain. As assessed by RP-HPLC, remarkably high pregnenolone and 7-dehydropregnenolone yields of 95 mg L^−1^ 48 h^−1^ could be achieved when using cholesterol or 7-DHC, respectively (Fig. 1b, c), which corresponds to a conversion of approximately 116 mg L^−1^ 48 h^−1^ (300 µM) of these substrates (Fig. 1f). The phytosterol β-sitosterol was converted to a lesser extent (Fig. 1d), with a conversion rate of 25 mg L^−1^ 48 h^−1^ (Fig. 1f). As illustrated in Fig. 1e, the addition of *Quillaja* saponin to the culture with a final concentration of 0.2% drastically increased the cholesterol conversion rate by 250%, demonstrating a new application of these naturally occurring surface-active glycosides for the permeabilization of bacterial membranes for steroidal compounds. The untransformed host strain MS941 was not able to metabolize cholesterol (Additional file 1: Figure S1). Whole-cell produced pregnenolone and 7-dehydropregnenolone were purified by preparative RP-HPLC and their structures confirmed by NMR spectroscopy against authentic standards.

### Identification of PHB bodies as main catalytic sites in recombinant *B. megaterium*

**Fig. 2:**
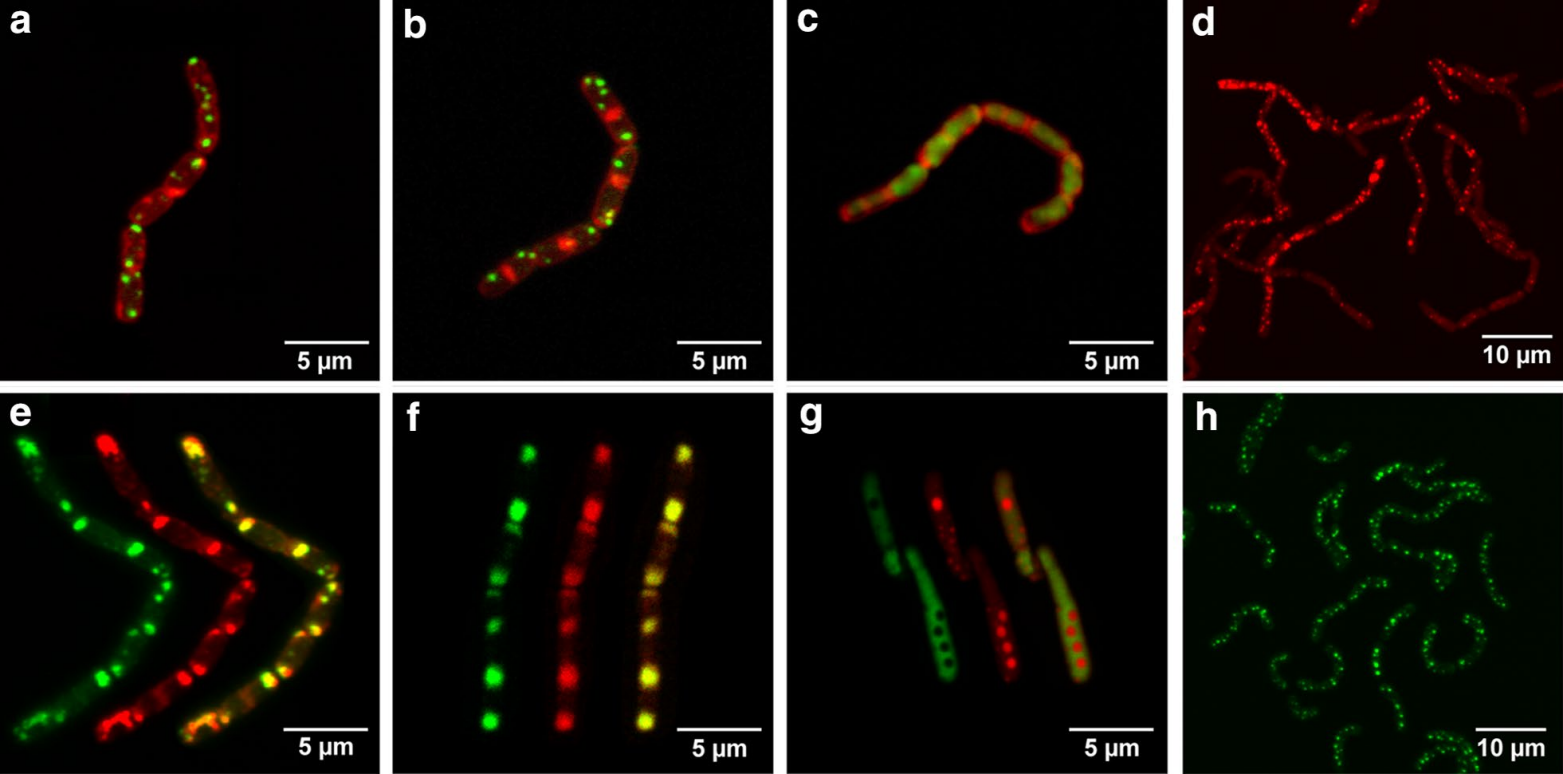
Localization of eGFP fused CYP11A1, AdR and Adx as well as 25-NBD cholesterol in cells of *B. megaterium* strain MS941 analyzed by confocal fluorescence microscopy. **a**–**c** CYP11A1eGFP, AdReGFP and AdxeGFP expressing cells, respectively, counterstained with FM 4-64. CYP11A1eGFP and AdReGFP form aggregates, while AdxeGFP is expressed in a soluble form. **d** Staining of untransformed cells with PHB specific dye Nile red. **e**–**g** Nile red staining of cells expressing CYP11A1eGFP, AdReGFP and AdxeGFP, respectively (merged images). From *left* to *right*: eGFP emission signal, Nile red emission signal, and overlay, showing overlapping signals in *yellow*. CYP11A1eGFP and AdReGFP are localized at the PHB granules, while AdxeGFP is present solely in the cytosol. **h** 25-NBD cholesterol accumulated in PHB bodies of cells. The number of single cells attached to one another via their peptidoglycane layer differs, leading to differently sized cell aggregates.

To evaluate the localization of the recombinantly produced proteins as well as the substrate in the cells of *B. megaterium*, we conducted confocal laser scanning microscopic (CLSM) studies. Firstly, fusion genes of either *cyp11A1*, *adx* and *adr* with *egfp* were constructed via SOE-PCR and cloned into vector pSMF2.1. *Bacillus megaterium* strain MS941 was transformed with the resulting plasmids pSMF2.1_CYP11A1eGFP, pSMF2.1_AdxeGFP or pSMF2.1_AdReGFP (Additional file 1: Figures S1–S3). Figures 2a–c illustrate the localization of the fusion proteins in the cells. To visualize a potential membrane localization of the recombinant proteins, an additional counterstaining with the fluorescent dye FM 4-64 [26] was carried out, which interacts with the polar phosphate groups of the outer leaflet of plasma membranes. While Adx exhibited a cytosolic distribution (Fig. 2c), the naturally membrane-associated proteins CYP11A1 and AdR were localized to distinct structures spread throughout the cells (Fig. 2a, b), which had a similar appearance to the PHB-bodies, monolayer coated hydrophobic aggregates of bioplastic serving as carbon storage, which can be specifically stained with the oxazone dye Nile red [27]. The cultivation of *B. megaterium* in TB-medium resulted in sufficient PHB granule formation for microscopic studies (Fig. 2d). Subsequently, fusion protein expressing strains were stained with Nile red. As shown in Fig. 2e, f, CYP11A1eGFP and AdReGFP localized to the PHB granules, providing evidence that both membrane proteins autonomously associate with these structure, without the addition of affinity tags to the proteins’ sequence. In contrast, the fusion between the soluble Adx and eGFP exhibited a cytosolic distribution and no association with the PHB granules (Fig. 2g).

To follow the fate of the substrate, we incubated cells of *B. megaterium* MS941 with the fluorescent cholesterol analog 25-[N-[(7-nitro-2-1,3-benzoxadiazol-4-yl)methyl]amino]-27-norcholesterol (25-NBD cholesterol) [28]. Again, this probe associated with the cells’ PHB granules (Fig. 2h), indicating that the substrate as well as the membrane proteins CYP11A1 and AdR are localized to these storage compounds.

### Deletion of PHA polymerase subunit PhaC: effect on cholesterol storage, PHB accumulation and in vivo conversion

In order to assess the role of the PHB-bodies in catalyzing the whole-cell conversion of steroids in recombinant *B. megaterium* in more detail, we aimed to delete the gene encoding the PHA polymerase subunit PhaC [29] to eliminate the formation of PHB-granules.

**Fig. 3:**
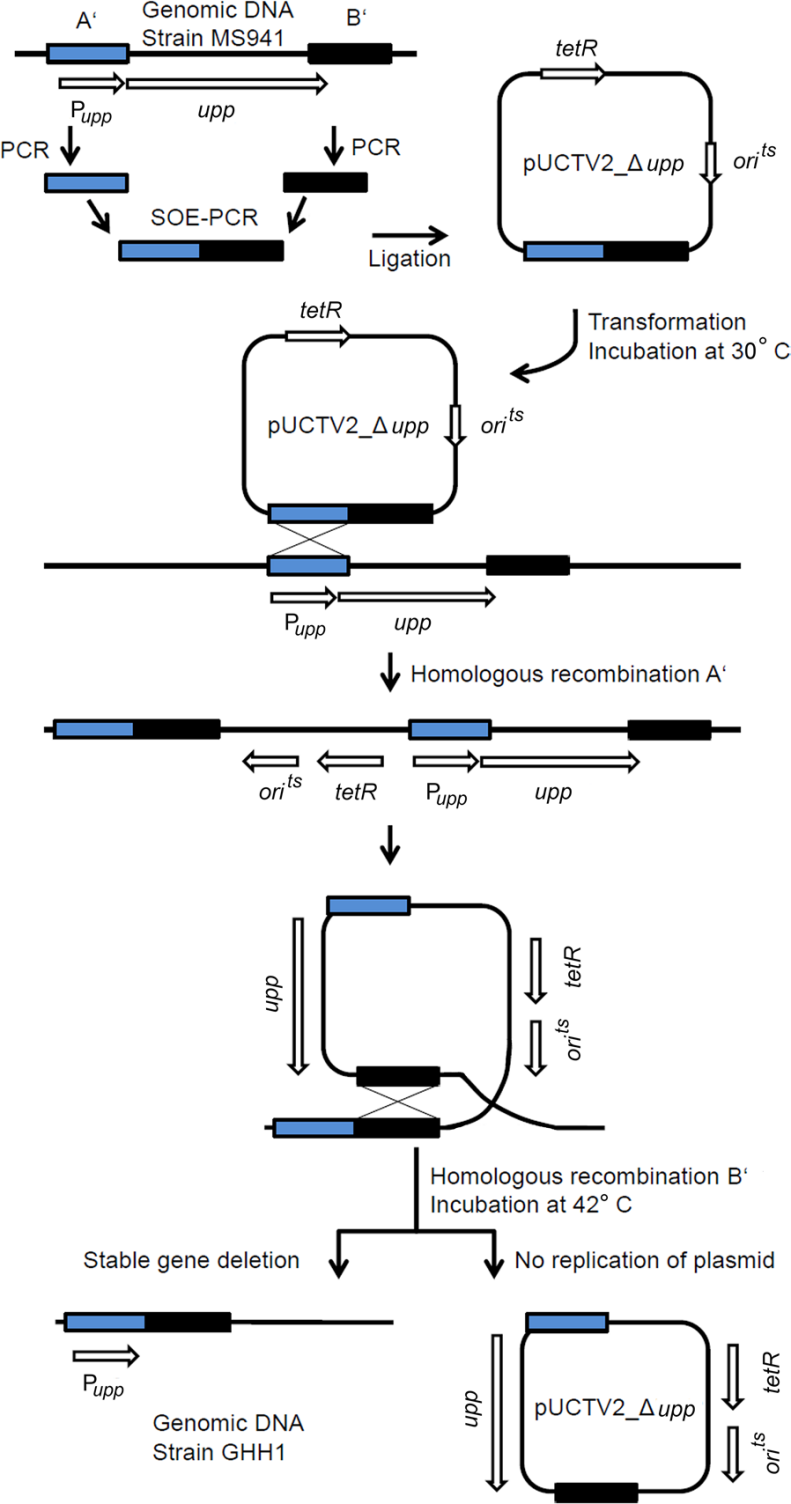
Schematic representation of *upp* deletion. Flanking genomic regions A′ and B′ of the target gene were amplified and fused via SOE-PCR. The resulting deletion construct was cloned into vector pUCTV2. *Bacillus megaterium* was transformed with the resulting plasmid pUCTV2_Δ*upp* and cultivated at 30°C. After two subsequent recombination events with each homologous fragment the deletion of *upp* was obtained. Incubation at the non-permissive temperature prevents replication of the plasmid containing the intact copy of the *upp* gene (*ori* ^*ts*^, temperature-sensitive origin of replication; *P*_*upp*_, promoter uracil phosphoribosyltransferase; *tetR*, tetracycline resistance gene; *upp*, uracil phosphoribosyltransferase gene).

**Fig. 4:**
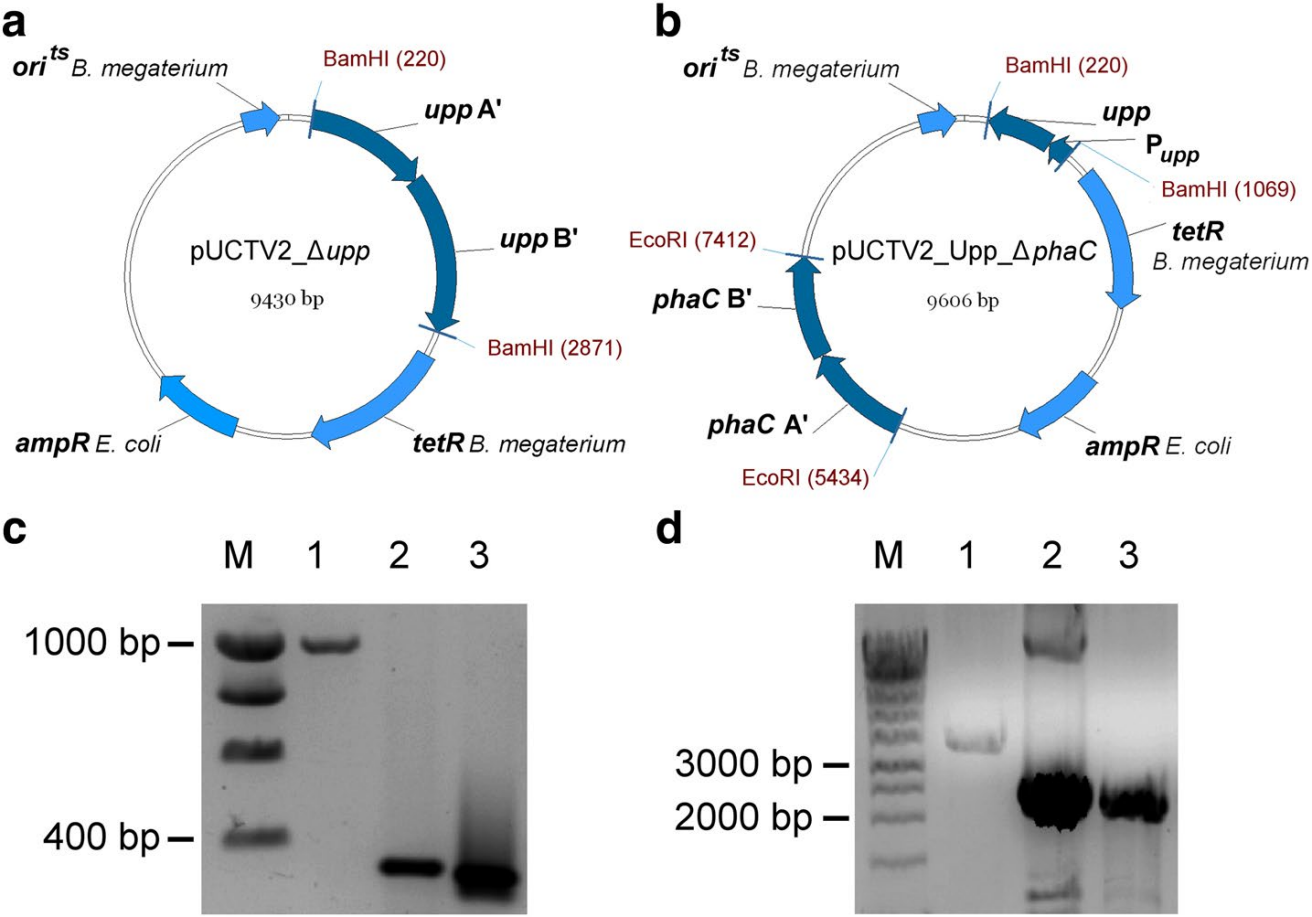
Deletion of the counterselection marker *upp* and the PHA polymerase subunit gene *phaC*. **a** Vector pUCTV2_Δ*upp* for the deletion of *upp*, containing flanking genomic regions upstream and downstream of the gene (*upp* A′ and *upp* B′). The temperature-sensitive origin of replication (*ori* ^*ts*^) prevents replication of the plasmid above temperatures of 42°C (*tetR*, tetracycline resistance gene; *ampR*, ampicillin resistance gene). **b** Vector pUCTV2_Upp_Δ*phaC* for the deletion of *phaC*, containing flanking genomic regions upstream and downstream of the gene (*phaC* A′ and *phaC* B′). It additionally contains the gene for the uracil phosphoribosyltransferase (*upp*) under the control of its natural promoter (P_*upp*_) which serves as a counterselection marker. **c** Agarose gel showing the PCR products using primers 9 and 10, flanking the *upp* gene and its promoter. Amplification with these primers results in DNA fragments of 865 bp size, if the *upp*-ORF is intact. The following templates were used: genomic DNA of MS941 (*lane 1*), pUCTV2_ Δ*upp* (*lane 2*) and genomic DNA of upp deletion strain GHH1 (*lane 3*). The resulting fragment contained the *upp* gene truncated by 600 bp (*lane 3*) compared to the full-length gene (*lane 1*) (*lane M*: *smartladder* DNA marker (Eurogentec). **d** Agarose gel showing the PCR products using flanking primers 11 and 14. These bind approximately 1,000 bp upstream and downstram of the target gene. Amplification with these primers results in DNA fragments of ca. 3,000 bp size, if the *phaC*-ORF is intact. The following templates were used: genomic DNA of strain GHH1 (*lane 1*), pUCTV2_Upp_Δ*phaC* (*lane 2*) and genomic DNA of strain GHH3 (*lane 3*). The resulting fragment contained the *phaC* gene truncated by 1,088 bp (*lane 3*) compared to the full-length fragment (*lane 1*) (*lane M*: *smartladder* DNA marker). Additional bands in *lane 2* stem from the plasmid used as DNA template.

In *B. megaterium*, deletion of genes can be achieved by transforming cells with a plasmid containing flanking regions of the target gene [30]. Two homologous recombination events with each flanking region are required to replace the target gene with the deletion construct (Fig. 3). Replication of the knockout plasmid can be prevented by cultivating cells at a non-permissive temperature, based on a temperature-sensitive origin of replication contained in this plasmid (Fig. 4a, b). However, this method alone proved to be inefficient for the deletion of *phaC*, since it resulted only in colonies still containing the full-length target gene. Therefore, a counter-selection system was established, based on a previously described method for *Bacillus subtilis*, making use of the uracil phosphoribosyltransferase (*upp*) gene [31]. The *upp* gene allows selection for cells that no longer possess the plasmid after the knockout procedure and are thus not sensitive to the highly toxic antimetabolite 5-fluorouracil (5-FU). Since *B. megaterium* MS941 naturally possesses this gene, at first a gene deletion mutant of this strain had to be created. Flanking regions of *upp* were cloned into the temperature-sensitive origin of replication-harboring vector pUCTV2 [30]. Cells of *B. megaterium* strain MS941 were transformed with the resulting vector pUCTV2_Δ*upp* and underwent the knockout procedure, as described in the “Methods” section. A colony was selected that was still able to grow on 5-FU containing agar. The deletion of the genomic *upp* ORF was confirmed by PCR (Fig. 4c) and the resulting strain was designated as GHH1. Then, flanking regions of the PHA polymerase subunit *phaC* were cloned into the vector pUCTV2_Upp containing the intact *upp* gene. Strain GHH1 was transformed with the resulting plasmid pUCTV2_Upp*_*Δ*phaC*. The deletion of the *phaC* ORF was successfully verified by utilizing a combination of the Upp-counterselection and a PCR-screening (Fig. 4d). The resulting strain was designated as GHH3.

**Fig. 5:**
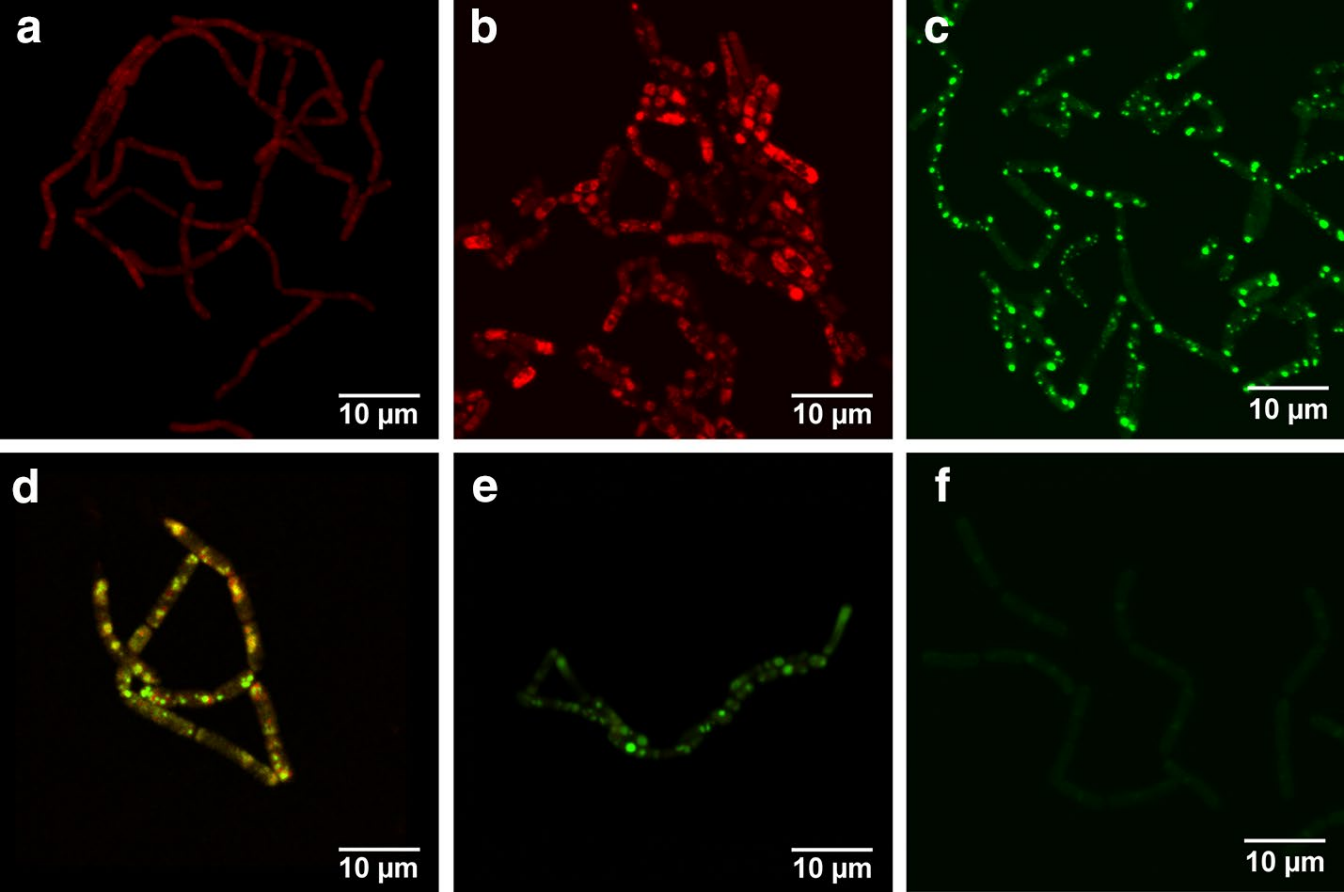
Effect of PHA polymerase subunit *phaC* knockout on morphology of strain GHH3. **a** Nile red stained Δ*phaC* strain GHH3 exhibits no accumulation of PHB. **b** PHB production is restored in cells after transformation with *phaC* containing plasmid pSMF3_RBCP. **c** Strain GHH3 transformed with pSMF2.1_CYP11A1eGFP. Expressed CYP11A1eGFP forms aggregates in the cell. **d**, **e** Expression of phaPeGFP in GHH1 and GHH3, respectively, both stained with Nile red (overlay). **f** Fluorescent probe 25-NBD cholesterol is not stored in cells of strain GHH3 lacking PHB.

Cells of strain GHH3 stained with Nile red completely lacked PHB accumulation (compare Fig. 5a with Fig. 2d). To exclude a possible unspecific gene deletion that could lead to an absence of PHB production, cells of strain GHH3 were transformed with plasmid pSMF3_RBCP (Additional file 1: Figure S5), encoding the genes of the PHB-synthesis operon: PHA polymerase subunit *phaR*, acetoacetyl-CoA reductase *phaB*, polyhydroxyalkanoic acid inclusion protein *phaP* and the genomically deleted gene *phaC*. As expected, the chromosomal deletion of *phac* was complemented, resulting in a restored PHB production (Fig. 5b).

The new strain GHH3 was transformed with pSMF2.1_CYP11A1eGFP. Surprisingly, the Nile red-stained cells of GHH3 expressing CYP11A1eGFP (Fig. 5c) displayed a similar distribution of the fusion protein in the bacterium compared with the PHB-producing strain GHH1 (Fig. 2e). We then expressed a fusion protein between eGFP and *B. megaterium*’s phasin phaP (Additional file 1: Figure S6), an amphiphilic protein associated with the PHB bodies’ monolayer, to assess whether the distribution of CYP11A1eGFP in strain GHH3 is an artifact stemming from the heterologous origin of the protein. As displayed in Fig. 5d, e, similar to CYP11A1eGFP, phaPeGFP exhibited an indistinguishable distribution in GHH1 and GHH3, both stained with Nile red, irrespective of PHB production.

As the disabled PHB production of the strain had no apparent effect on the overexpression of CYP11A1, we subsequently incubated strain GHH3 with the substrate analog 25-NBD cholesterol. The microscopic analysis revealed that the cells had lost the ability to store the sterol when no PHB was present (Fig. 5f).

**Fig. 6:**
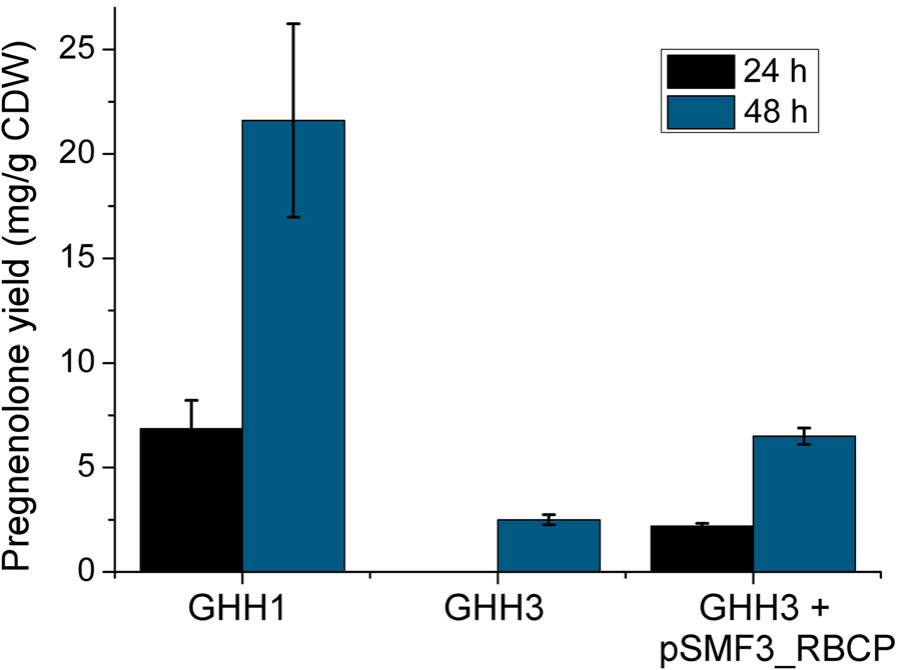
Effect of disabled PHB formation on cholesterol conversion activity. Pregnenolone formation in PHB-producing strain GHH1 and PHB-devoid strain GHH3, both transformed with pSMF2.1_SCCAA. After 24 h no pregnenolone could be detected in cultures of strain GHH3. The product formation after 48 h was decreased by 88% in comparison to strain GHH1. As a control strain, GHH3 was co-transformed with pSMF3_RBCP resulting in a restored PHB production and cholesterol conversion activity. *Error bars* indicate standard deviation of triplicate biological experiments.

Lastly, we investigated whether the impaired substrate storage in absence of PHB in *B. megaterium* cells has an effect on the in vivo whole-cell cholesterol conversion rate. We compared strain GHH3 with GHH1 and pSMF3_RBCP-containing GHH3, each one being transformed with pSMF2.1_SCCAA, encoding the genes for CYP11A1, Adx and AdR. Figure 6 illustrates the pregnenolone yield for each strain normalized to the cell dry weight, to exclude a potential effect of the gene deletion on the growth of *B. megaterium* cells. The absence of PHB in cells of GHH3 led to no detectable product formation after 24 h and an 88% decreased pregnenolone yield after 48 h compared with GHH1. The capacity to convert cholesterol was partly restored in strain GHH3 transformed with the complementing PhaC encoding plasmid pSMF3_RBCP.

## Discussion

With an annual worldwide production exceeding 1,000,000 tons, steroid drugs are the second most marketed medical agents next to antibiotics [32]. The biotechnological steroid production is one of the best examples of the successful combination of microbial conversions and chemical reaction steps [1], since some specific modifications of the steroid skeleton cannot be performed by the latter. Examples of such reactions are stereo- and regioselective hydroxylations, dehydrogenations, and the steroid side-chain cleavage presented in this paper. Although there are many microorganisms of different taxa, which are able to convert cholesterol or phytosterols to androstenedione, androstadienedione or other C19-steroids, the efficient microbial conversion of these substrates to pregnenolone has not been described so far [33, 34]. The most abundant steroid in vertebrates, cholesterol, can be obtained in huge amounts by extraction of wool grease or bovine spinal cords [35].

In this study we developed a recombinant whole-cell catalyst that efficiently cleaves the side–chain of sterols, converting the substrates cholesterol and β-sitosterol to pregnenolone, and 7-dehydrocholesterol to 7-dehydropregnenolone. In order to perform these reactions, the Gram-positive bacterium *B*. *megaterium* strain MS941 served as a host for the expression of the bovine side-chain cleavage enzyme CYP11A1 and its redox partners Adx and AdR under control of the inducible strong xylose promoter. The resulting recombinant strain converted each of the three substrates in our study to one specific product (Fig. 1b–d). NMR-spectroscopy confirmed that the expected products pregnenolone or 7-dehydropregnenolone were formed with no visible side-product formation. The addition of *Quillaja* saponin, a mixture of amphiphilic glycosides obtained from the ‘soap bark tree’ *Quillaja saponaria* [36] greatly improved the sterol conversion rate (Fig. 1e). It has been demonstrated before that these compounds increase cholesterol solubility in aqueous solutions by micelle formation [37] and are able to form pores in biological membranes in complex with cholesterol [38]. We propose that saponins can facilitate the transport of cholesterol into the whole-cell system of *B. megaterium* without having detrimental effects on the cells, since cultures were still able to grow and convert cholesterol up to 48 h after addition of the additive in the early exponential phase, while maintaining their morphological integrity. Once the concentration of the additive was optimized (Fig. 1e), 300 µM of cholesterol were completely converted to pregnenolone in 48 h. These values have been reached under simple discontinous shake-flask cultivation and already exceed the pregnenolone concentration described for high-cell density cultivation of recombinant *S. cerevisae* (~60 µM 48 h^−1^; [6]). Comparable systems for pregnenolone production do not exist. 7-DHC was converted with an equal rate, while β-sitosterol conversion was considerably lesser (Fig. 1f). The reduced conversion rate of the latter is in congruence with published experiments using a reconstituted system containing purified CYP11A1, AdR and Adx displaying a 66% lower turnover number (kcat) of CYP11A1 for β-sitosterol (and 46% for campesterol) compared with cholesterol [39]. This may be attributed to the more branched side-chain of β-sitosterol, which possibly slows down its entry into the active site of CYP11A1 and its movement during the three-step side-chain cleavage reaction. Site-directed mutagenesis of the CYP11A1 gene to generate a protein providing more space for the bulky substrate might solve this problem in the future.

In order to elucidate the macromolecular basis for the efficient transfer and conversion of these hydrophobic substrates in *B. megaterium,* we firstly analyzed the localization of the recombinant proteins. *B. megaterium* strains were generated producing C-terminally tagged eGFP fusion proteins of CYP11A1, AdR and Adx. CLSM analysis revealed that AdxeGFP was localized in the cytosol (Fig. 2g). This is in accordance with its function as a soluble protein shuttle, transferring electrons from AdR to CYP11A1. Surprisingly, CYP11A1 and AdR were shown to be localized at the PHB granules (Fig. 2e, f), as demonstrated by Nile red staining.

PHB granules accumulate in the bacterium upon excessive carbon supply in the medium and can be mobilized as a source of energy when carbon is limited [40]. The granules’ surface appears to provide an optimal environment for the functional association of the mammalian membrane proteins CYP11A1 and AdR. Although this result was unexpected, the principle association to a phospholipid monolayer is in accordance with their nature as membrane proteins.

We further investigated the localization of the substrate in cells of *B. megaterium* utlizing the fluorescent analog 25-NBD cholesterol. As in the case of AdReGFP and CYP11A1eGFP, the probe accumulated in the PHB granules (Fig. 2h). Concluding from these findings, we proposed that *B. megaterium* can efficiently take up the substrate under the applied biotransformation conditions and that the highly hydrophobic core of the PHB granules is able to store cholesterol, which is subsequently converted by CYP11A1, associated with the surrounding monolayer.

To provide further evidence for this assumption, we aimed to disable the PHB granule production by deleting the large PHA polymerase subunit encoding gene *phaC*, applying a new counter-selection system for *B. megaterium*. The resulting strain GHH3 was unable to produce PHB, as demonstrated by Nile red staining. The PHB production could be restored by transforming this strain with a plasmid encoding the PHB operon including *phaC*, although the shape of the cells and granula differed from the wild type strain GHH1, possibly due to the overexpression of the PHB granules-synthesizing proteins and the increased PHB accumulation.

Contrary to our expectation that CYP11A1 tagged with eGFP would now be localized at the bacterium’s membrane, the fusion protein expressed in the PHB-devoid strain GHH3 still exhibited a similar aggregation pattern compared with strain GHH1, although no PHB granules should be present in the cells (Fig. 5c; cross-sections through cells of both strains are displayed in Additional file 1: Figure S7). According to the three existing models, activity of the PHA polymerase is necessary for the biogenesis of PHB granules in microorganisms [41–43]: the ‘micelle’ model suggests that PHB is produced in the cytosol and the monolayer coats the PHB when a certain size is reached, the ‘budding’ model states that PHB is produced in the intermembrane space, leading to a budding off the membrane and lastly the ‘scaffold’ model proposes that the PHB synthesis is initiated from the bacterial nucleoid, to which the required proteins are attached. We repeated the localization experiment overexpressing *B. megaterium*’s chromosomally encoded phasin phaP, a natural main constituent of the PHB granules’ monolayer responsible for size regulation, to assess whether the aggregation of CYP11A1eGFP is an artificial effect due to the protein’s heterologous nature. However, similar to CYP11A1eGFP, phasineGFP was expressed in aggregates in both GHH1 and GHH3 (Fig. 5d, e). It remains to be elucidated if the observed protein structures are ‘hollow’ PHB bodies, proteinaceous inclusion bodies or self-oligomerized protein complexes, e.g. by investigating the localization of other naturally PHB monolayer associated proteins. Self-oligomerization of the eGFP tag can be excluded, since AdxeGFP exhibits a cytosolic distribution.

In contrast to the CYP11A1 expression, accumulation of the cholesterol analog 25-NBD cholesterol inside the cells of PHB-lacking strain GHH3 was not observed, indicating that substrate storage and uptake is the crucial factor for the efficiency of the whole-cell system (Fig. 5f).

To substantiate this finding, the in vivo cholesterol conversion activity with strains GHH1and GHH3 expressing CYP11A1 and its redox partners was assessed (Fig. 6). The pregnenolone yield for the PHB-devoid strain was drastically decreased, but could be increased again after complementing the PHB formation in strain GHH3 by co-transformation with a plasmid encoding the PHB synthesis operon. Pregnenolone levels did not reach those of strain GHH1, which could be explained by lower expression levels of CYP11A1, Adx and AdR, as well as decreased NADPH availability due to the overexpression of the PHB synthesizing operon. In conclusion, the strongly impaired substrate storage observed by fluorescence microscopy is in congruence with the diminished cholesterol conversion rate in *B. megaterium* cells lacking PHB production.

Overall, our results provide evidence that the efficient conversion of cholesterol in the recombinant *B. megaterium* whole-cell system is based on the natural ability of the microorganism to form subcellular scaffolds in form of PHB granules. The core of these granules serves as storage for hydrophobic sterols, whereas the surface layer can host mammalian membrane proteins in an active form. This system was applied for the conversion of the hydrophobic substrates cholesterol and β-sitosterol to pregnenolone and 7-dehydrocholesterol to 7-dehydropregnenolone, both pharmaceutically interesting starting compounds for further steroid hormone production, by the side-chain cleaving cytochrome P450 CYP11A1. Taken together, *B. megaterium* is a particularly promising host for the expression of further cholesterol-metabolizing membrane proteins such as CYP27A1, CYP3A4 or CYP46A1 for biotechnological applications or the elucidation of new products. In addition, it could generally serve as an expression platform for membrane proteins, such as microsomal Cytochromes P450 and be suitable for the bioconversion of hydrophobic substrates with an affinity for the PHA synthesized by this bacterium.

## Conclusion

**Fig. 7:**
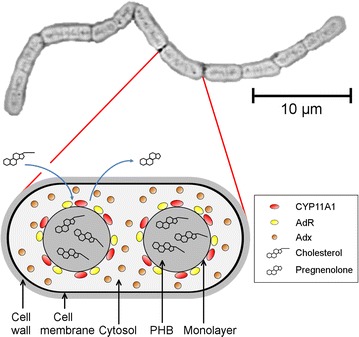
Summary illustration. In cells of recombinant *B. megaterium*, the membrane proteins CYP11A1 and AdR are localized to the PHB granule-surrounding monolayer. Adx is localized to the cytosol, serving as an electron shuttle between CYP11A1 and AdR. Cholesterol is stored inside the PHB core and can be delivered to CYP11A1. After undergoing the side-chain cleavage, the product pregnenolone is released from the cell.

Cholesterol is the precursor molecule of all steroid hormones, encompassing glucocorticoids, mineralocorticoids and sex steroids. Due to its extremely low solubility in water, an efficient microbial conversion has not been possible so far. In the present paper we describe a whole-cell system based on the soil bacterium *B. megaterium*, which, by recombinantly expressing the side-chain cleaving cytochrome P450 CYP11A1 and its redoxpartners, is able to efficiently convert cholesterol to pregenenolone, the central compound of steroidogenesis. We demonstrate that the microorganism’s PHB granules, aggregates of bioplastic coated with a protein/phospholipid monolayer, are crucial for the high conversion rate by serving as substrate storage. Furthermore, we provide evidence that the natural membrane proteins CYP11A1 and its redox partner AdR are attached to the granules’ monolayer, implying that these structures can host eukaryotic membrane proteins in a properly folded state (Fig. 7). This microbial system allows the targeted microbial production of steroid hormones starting from the abundantly available substrate cholesterol or analogs.

## Methods

### Molecular cloning

Sequences of the bovine genes encoding mature CYP11A1 (mutant K193E [44] based on NCBI Reference Sequence: NP_788817.1), AdR (NP_777116.1) and Adx (NP_851354.1) were codon-optimized for *B. megaterium* (http://www.jcat.de) and synthesized by Geneart AG (Regensburg, Germany). The resulting genes were cloned into shuttle vector pSMF2.1 [45] by using SpeI/MluI, KpnI/SacI and BsrGI/SphI restriction sites, respectively, resulting in plasmid pSMF2.1_SCCAA. All three genes are located in an operon under the control of the strong inducible promoter P_*xylA*_. Each gene contains its own ribosomal binding site. In *E. coli*, the ß-lactamase is produced, conferring ampicillin resistance. In *B. megaterium*, the tetracycline efflux pump is produced, conferring tetracycline resistance. Plasmids pSMF2.1_CYP11A1eGFP, pSMF2.1_AdReGFP and pSMF2.1_AdxeGFP encoding CYP11A1, AdR and Adx with a C-terminally fused eGFP tag (Additional file 1: Figures S2–S4) were constructed by SOE-PCR using the respective primers 2a/3a, 2b/3b and 2c/3c in combination with primers 1 and 4 and pSMF2.1_SCCAA and pEGFP-C1 (clontech) as templates. The resulting fused genes were cloned into pSMF2.1 with restrictions sites SpeI and MluI. The fusion gene encoding phaPeGFP (Additional file 1: Figure S6) was constructed by SOE-PCR using primers 21-24 with genomic *B. megaterium* DNA pEGFP-C1 (clontech) as templates. The resulting fragment was cloned into pSMF2.1 using PacI and SpeI restriction site resulting in vector pSMF2.1_phaPeGFP. Vector pUCTV2_Δ*upp* was constructed by cloning flanking regions A’ and B’ into vector pUCTV2 via BamHI sites. The deletion construct was created by SOE-PCR using genomic DNA of *B. megaterium* strain MS941 as template and primers 5, 6, 7 and 8. Vector pUCTV2_Upp_Δ*phaC* was constructed by cloning the full-length *upp* gene and its natural promoter into pUCTV2 using a BamHI site, after amplifying the fragment by PCR using genomic DNA of *B. megaterium* strain MS941 and primers 9 and 10. The deletion construct containing A’ and B’ was amplified and fused by SOE-PCR using genomic DNA as template and primers 11, 12, 13 and 14, then ligated into plasmid pUCTV2_Upp via the EcoRI restriction site. Vector pSMF3_RBCP was constructed by ligating two PCR fragments sequentially into the vector pMGBm19 [46] via the restriction sites XhoI, MluI and SacI. The first fragment comprising the operon of *phaR*, *phaB* and *phaC* under control of the natural promoter P_*phaR*_ and the second fragment comprising the *phaP* gene were amplified using genomic DNA of *B. megaterium* strain MS941 and primers 15, 16, 17 and 18 resulting in the new operon P_*phaR*_-*phaR*-*phaB*-*phaC*-*phaP* (Additional file 1: Figure S5).

Sequences of all primers are listed in Additional file 1: Table S1. Strains and plasmids used in this study are listed in Additional file 1: Tables S2, S3. All cloning experiments were performed with *E. coli* strain Top10 (Invitrogen). Plasmids were prepared using Nucleospin Plasmid QuickPure kit (Macherey & Nagel). Genomic DNA was extracted by Genomic DNA Extraction kit (nexttec). Ligations were carried out using FastLink DNA Ligase kit (Epicentre Biotechnologies). All restriction enzymes were purchased from New England Biolabs. *Bacillus megaterium* cells were transformed according to the PEG-mediated protoplast transformation method [47].

### *B. megaterium* cultivation conditions

Either LB- (25 g/L, Becton–Dickinson) or TB- (24 g/L yeast extract, 12 g/L tryptone, 0.4% glycerol, 100 mM potassium phosphate buffer, pH 7.4) media were used for the cultivation of *B. megaterium*. For the preculture, 50 mL of medium either containing 10 µg/mL tetracycline (cells harboring a variant of pSMF2.1) or 10 µg/mL chloramphenicol (cells harboring a variant of pSMF3) were inoculated with cells from an agar plate or glycerol stock. For the main culture, 50 mL of medium containing 10 µg/mL of the required antibiotic were inoculated with 500 µL of the preculture. The main culture was grown until an optical density of ~0.4 was reached, protein expression was induced by addition of 0.25 g xylose dissolved in 1 mL distilled water. For whole-cell conversion experiments, substrates were dissolved in a 45% 2-hydroxypropyl-β-cyclodextrin/4% *Quillaja* saponin-solution. 2.5 mL of the solution were added to the culture directly after protein induction. A final substrate concentration of 300 µM was used for all conversion experiments. Cholesterol from sheep wool (purity ≥99%), 7-dehydrocholesterol (≥98%), β-sitosterol from plant extracts (purity ≥70%, containing residual campesterol), 2-hydroxypropyl-β-cyclodextrin (average molecular weight ~1,460) and *Quillaja* saponin (Sapogenin content ≥10%) were purchased from Sigma-Aldrich.

### Sample treatment and RP-HPLC analysis

Steroids without intrinsic absorption were converted into their Δ_4_-3-keto-derivatives prior to RP-HPLC analysis, to allow photometric detection at 240 nm. 1 mL culture samples were boiled in water for 1 min. 20 µL cholesterol oxidase solution (5 mg cholesterol oxidase (Calbiochem) and 5 mg Na-cholate dissolved in 5 mL 50 mM HEPES buffer pH 7, containing 0.05% Tween-20) were added and the sample was incubated at 37°C with 1,000 rpm shaking for 1 h. For RP-HPLC analysis, the sample was extracted twice with 1 mL ethylacetate and the extract was dissolved in acetonitrile. Samples were analyzed isocratically with pure acetonitrile using a NUCLEODUR 100-3 C_18_ column (Macherey & Nagel). Samples containing cholestenone and β-sitostenone were detected at 240 nm wavelength, 7-DHC at 280 nm. A flow rate of 1 mL/min was applied. Progesterone yield was calculated by using a progesterone calibration curve. 7-Dehydropregnenolone yield was determined by dividing product peak area by total substrate/product peak area. To purify pregnenolone for NMR analysis after whole-cell conversion, 200 mL culture medium were extracted and separated using a 250/8 NUCLEODUR 100-3 C_18_ column and the product collected.

### Cell staining and confocal fluorescence microscopy

*Bacillus megaterium* was cultivated under the same conditions as for the in vivo conversion experiments. Prior to staining, culture samples were washed and centrifuged twice to diminish background fluorescence. 20 µL of cell suspension were spread on coverslips and fixed by air drying. For Nile red (Sigma-Aldrich) staining, drops of a solution of 1 mg/mL Nile red dissolved in ethanol were added to the coverslips, incubated for 10 s and rinsed with ethanol. For FM 4-64 (Invitrogen) staining drops of a solution of 1 mg/mL FM 4-64 dissolved in distilled water were added to the coverslips, incubated for 10 s and rinsed with water. Samples were analyzed with a LSM 510 confocal microscope (Zeiss). Excitation and emission maxima for eGFP, Nile red and FM 4-64 are as follows (in nm): 488/509, 552/636 and 558/734. Cells were magnified 1,000× using immersion oil and digitally zoomed in, if necessary.

### Gene deletion in *B. megaterium*

To delete the *upp* gene, *B. megaterium* MS941 was transformed with the plasmid pUCTV2_Δ*upp* containing the deletion construct. 50 mL LB medium containing 10 µg/mL tetracycline were inoculated with transformed colonies and incubated at 30°C and 180 rpm for 16 h. Culture samples were diluted 1:10 and spread out on minimal medium agar plates, based on modified Spizizen’s salts [48], and incubated at 30°C for 16 h. Colonies were then replica plated and incubated at 42°C for 16 h. Colonies containing a genomic deletion of the *upp* gene were able to grow on minimal agar plates containing 1 µM 5-FU. The truncation of the gene was verified by PCR, using primers 9 and 10. The resulting strain was named GHH1. The *phaC* gene was knocked out by transforming cells of strain GHH1 with plasmid pUCTV2_Upp_Δ*phaC*. 50 mL LB medium containing 10 µg/mL tetracycline were inoculated with transformed colonies and incubated at 30°C and 180 rpm for 16 h. Culture samples were diluted 1:10, spread out on minimal medium agar plates and incubated at 30°C for 16 h. Colonies were replica plated on minimal agar plates containing 1 µM 5-FU and incubated at 42°C for 16 h. The resulting grown colonies either contained the full-length or truncated form of the *phaC* gene. Absence of PHB production was confirmed by Nile red staining and the gene deletion verified by PCR using primers 11 and 14. The resulting strain was designated as GHH3.

## Additional file

Additional file 1. Supplemental data containing plasmid maps, list of primers and strains and additional results.
