# Supplementary material for: Functionalized PHB granules provide the basis for the efficient side-chain cleavage of cholesterol and analogs in recombinant Bacillus megaterium
Source: Microb Cell Fact. 2015 Jul 29;14:107. doi: 10.1186/s12934-015-0300-y (PMC4517628; doi:10.1186/s12934-015-0300-y)
Supplement: Additional file 1. — Supplemental data containing plasmid maps, list of primers and strains and additional results. [file 12934_2015_300_MOESM1_ESM.docx]

Supplemental Data


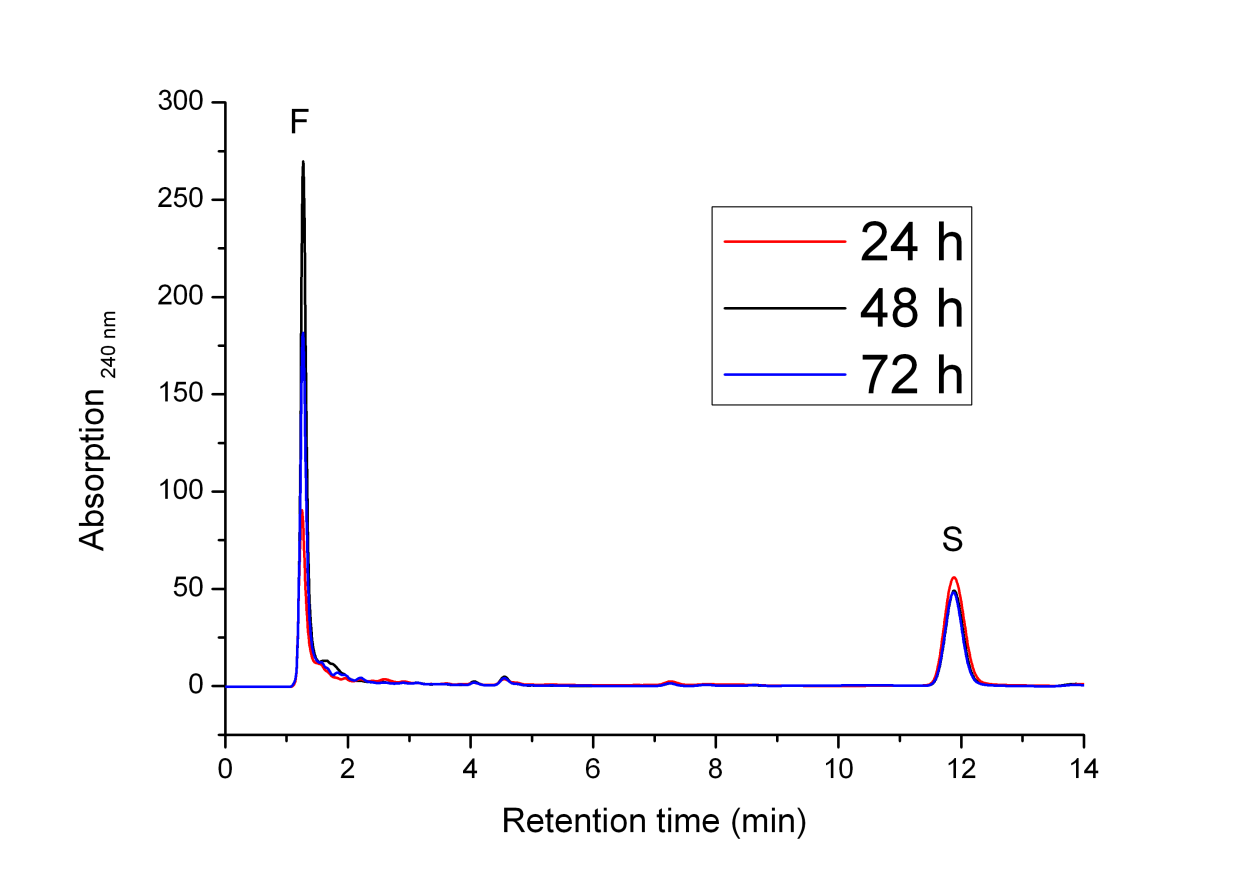


**Figure S1 Incubation of *B. megaterium* strain MS941 with cholesterol.** No significant conversion of cholesterol could be observed after up to 3 days (S: cholesterol, F: flow-through peak).


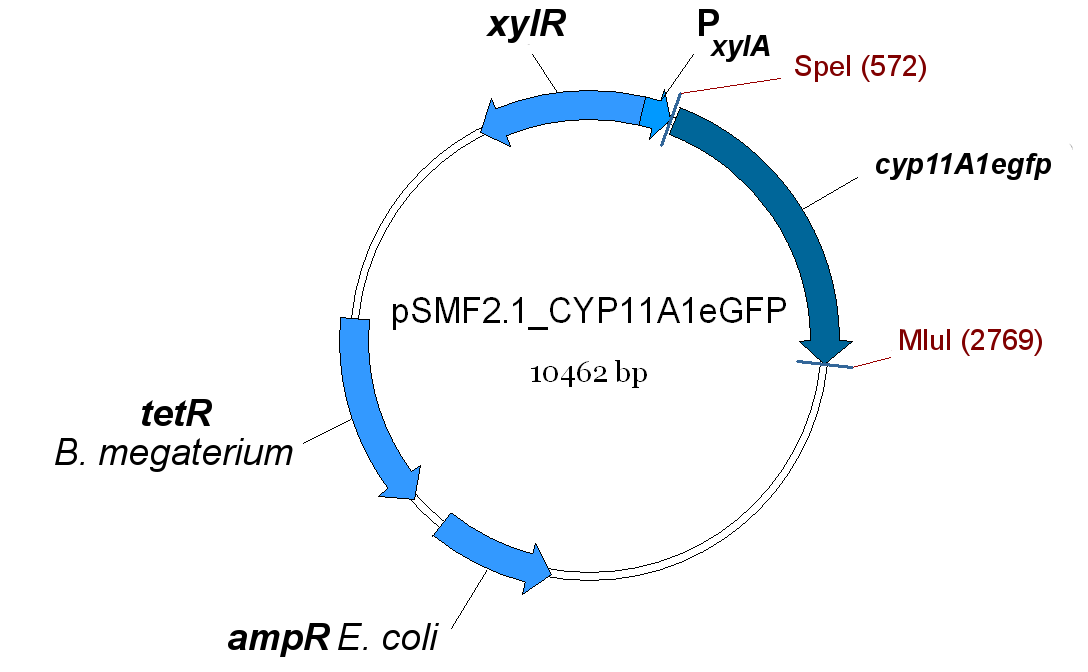


Figure S2 Map of plasmid pSMF2.1_CYP11A1eGFP. The gene encoding CYP11A1eGFP is cloned downstream of the xylose-inducible promoter using SpeI and MluI restriction sites (P*xylA*: promoter of the xylose utilization operon, *xylR*: xylose repressor, *tetR*: tetracycline resistance gene , *ampR*: ampicillin resistance gene).


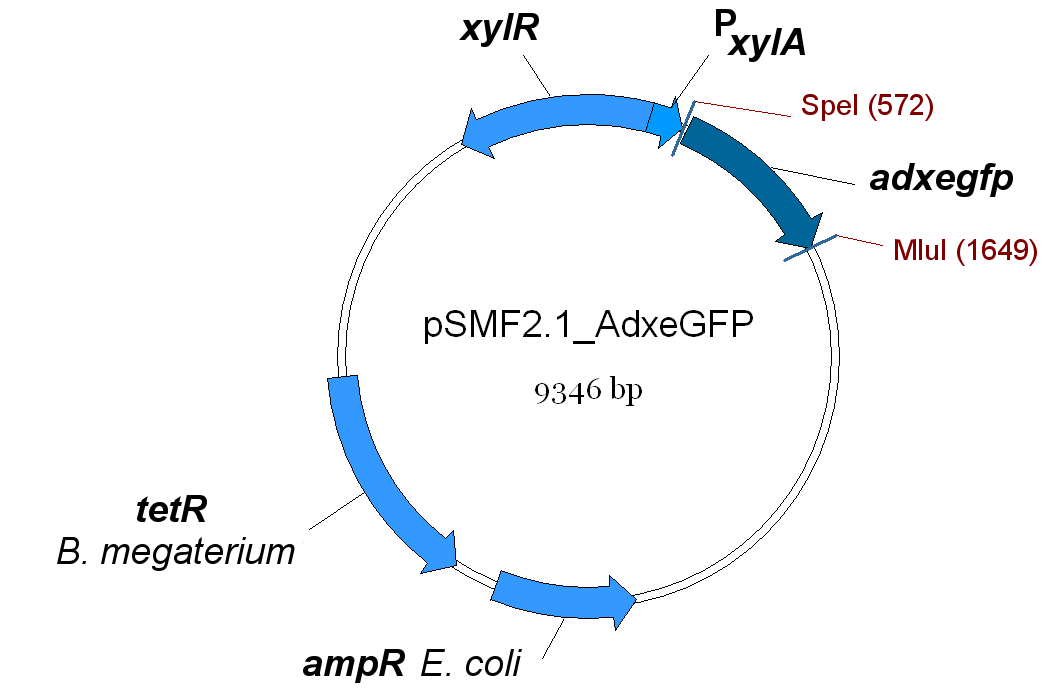


Figure S3 Map of plasmid pSMF2.1_AdxeGFP. The gene encoding AdxeGFP is cloned downstream of the xylose-inducible promoter using SpeI and MluI restriction sites (P*xylA*: promoter of the xylose utilization operon, *xylR*: xylose repressor, *tetR*: tetracycline resistance gene , *ampR*: ampicillin resistance gene).


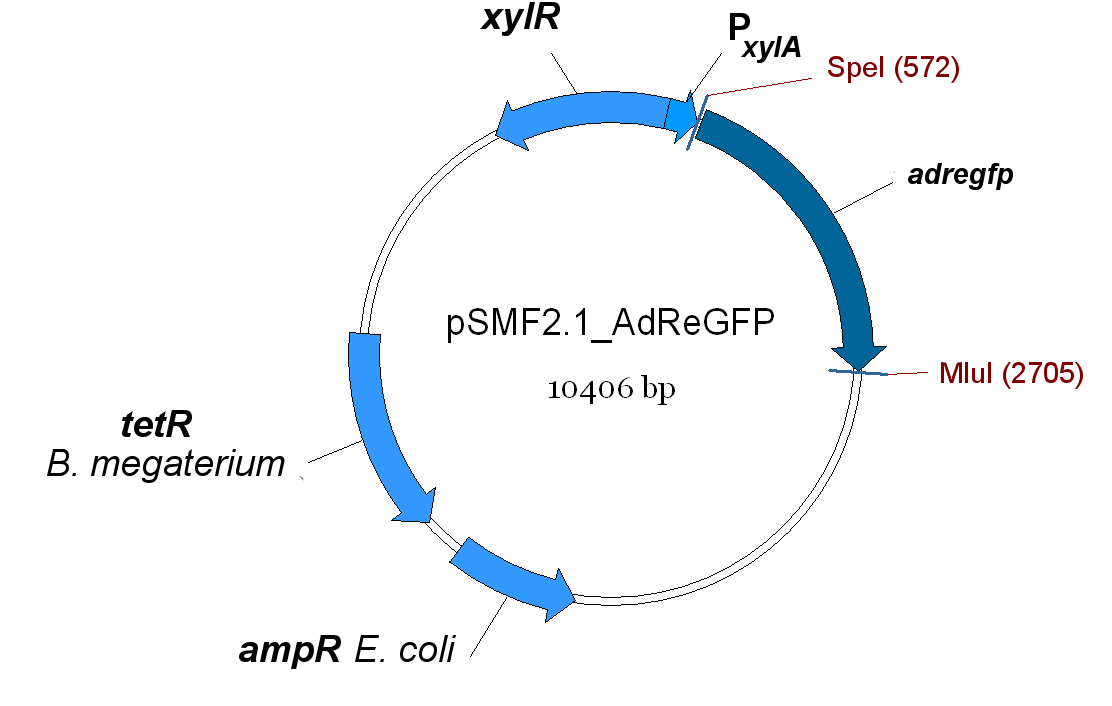


Figure S4 Map of plasmid pSMF2.1_AdReGFP. The gene encoding AdReGFP is cloned downstream of the xylose-inducible promoter using SpeI and MluI restriction sites (P*xylA*: promoter of the xylose utilization operon, *xylR*: xylose repressor, *tetR*: tetracycline resistance gene , *ampR*: ampicillin resistance gene).


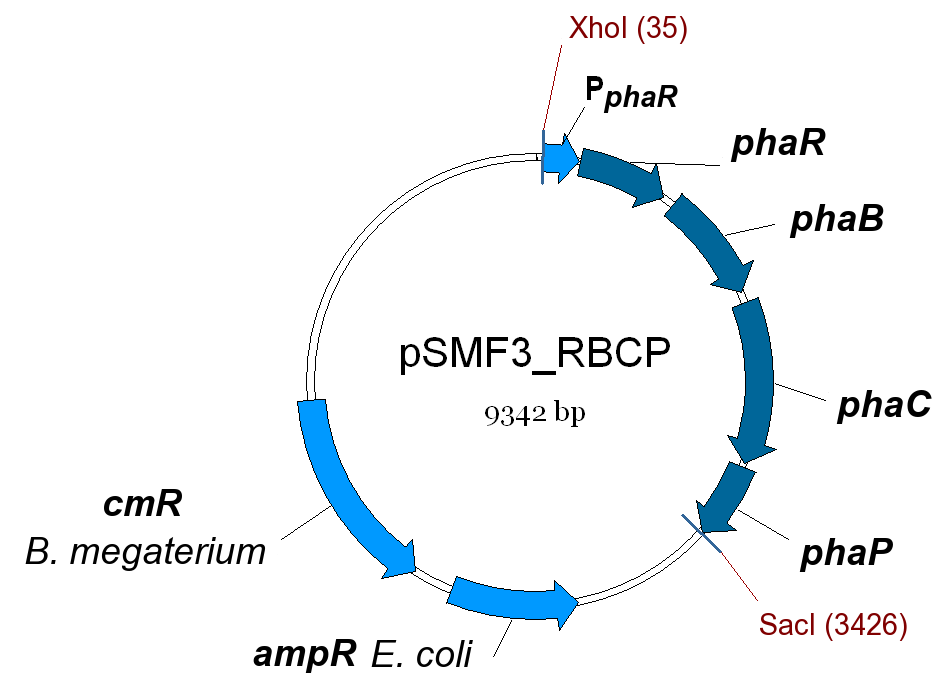


Figure S5 Map of plasmid pSMF3_RBCP. Genes for PhaR (polyhydroxyalkanoic acid synthase, PhaR subunit), PhaB (acetoacetyl-CoA reductase), PhaC (polyhydroxyalkanoic acid synthase, PhaC subunit) and PhaP (polyhydroxyalkanoic acid inclusion protein) are cloned downstream of the the natural *phaR*-gene promoter P*phaR* using XhoI and SacI restriction sites (*cmR*: chloramphenicol resistance gene, *ampR*: ampicillin resistance gene).


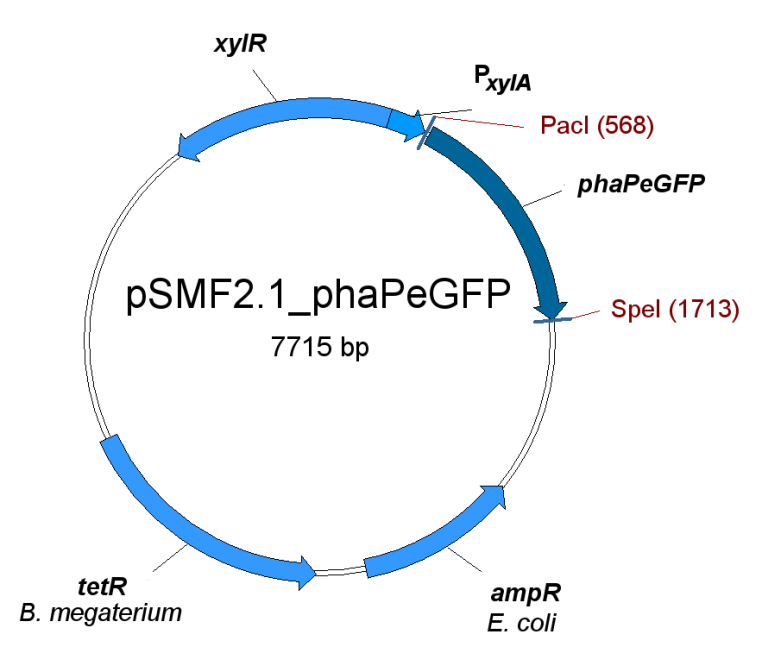


Figure S6 Map of plasmid pSMF2.1_phaPeGFP. The gene encoding phaPeGFP is cloned downstream of the xylose-inducible promoter using PacI and SpeI restriction sites (P*xylA*: promoter of the xylose utilization operon, *xylR*: xylose repressor, *tetR*: tetracycline resistance gene , *ampR*: ampicillin resistance gene).


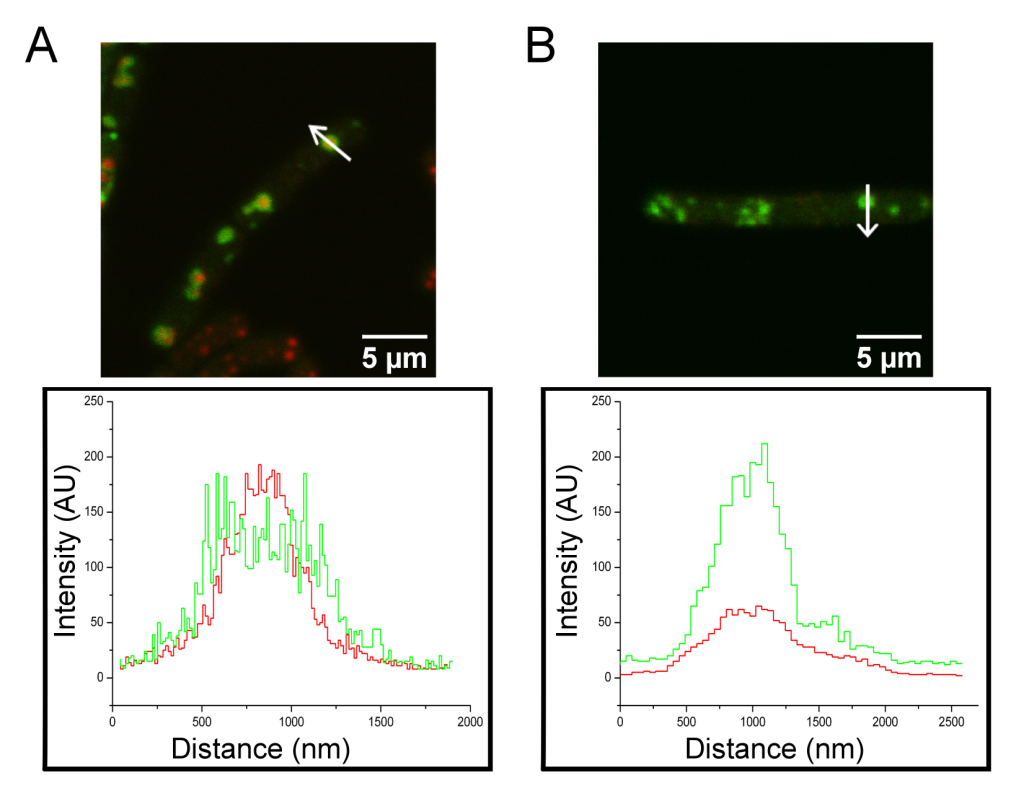


Figure S7 Cross-section through Nile red-stained cells of GHH1 and GHH3 expressing CYP11A1eGFP. (A) Emission signals of eGFP are confined to the PHB-surrounding membrane (green curve) in confocal cross-sections of cells of strain GHH1 expressing CYP11A1eGFP, stained with Nile red. A high Nile red emission signal is detectable in the core of the PHB body (red curve). (B) Only a slight background Nile red emission signal is detectable in cells of strain GHH3 lacking PHB (red curve), possibly from a cross-talk with eGFP emission. CYP11A1eGFP still accumulates as aggregates (green curve).

Table S1 List of primer sequences.

| **Primer number** | **Primer name** | **Sequence (5‘ --> 3‘)** |
| --- | --- | --- |
| 1 | AdxEYFP_1 | ACTAGTAATCAAGGAGGTGAATATACAATG |
| 2a | CYPEYFP_2 | AGCTCCTCGCCCTTGCTCACCATAGCTTGAGGAGGATCTTGATTAAAAGG |
| 2b | AdREYFP_2 | CAGCTCCTCGCCCTTGCTCACCATGTGGCCAAGTAAACGAAGCATTTC |
| 2c | AdxEYFP_2 | CAGCTCCTCGCCCTTGCTCACCATTGGTACTCGAACAGTCATATTG |
| 3a | CYPEYFP_3 | TTTAATCAAGATCCTCCTCAAGCTATGGTGAGCAAGGGCGAGGAGCTG |
| 3b | AdREYFP_3 | GAAATGCTTCGTTTACTTGGCCACATGGTGAGCAAGGGCGAGGAGCTG |
| 3c | AdxEYFP_3 | CAATATGACTGTTCGAGTACCAATGGTGAGCAAGGGCGAGGAGCTG |
| 4 | FP_B2 | GCATACGCGTTTATTACTTCTACAGCTCGTCCATGCC |
| 5 | Upp_A_for | TATCAGGATCCgtagctaatagcatcacaaatacgtttagaagc |
| 6 | Upp_B_rev | gagatatcaatgggcaaagtatacgtatttgatcgggatcgtttattcggtacaaaataatttgtagatt |
| 7 | Upp_C_for | aatctacaaattattttgtaccgaataaacgatcccgatcaaatacgtatactttgcccattgatatctc |
| 8 | Upp_D_rev | TATCAGGATCCcattagttgtttctccatcaaaatcctctcc |
| 9 | Upp_for_*BamH*I | TATCAGGATCCTTA TTT TGT ACC GAA TAA ACG ATC CCC |
| 10 | Upp_rev_*BamH*I | TATCAGGATCCGAG AGA CAG GCC TTT GTG C |
| 11 | ko_phaC_A | atgcGAATTCAGGCTCAGCTTTTAGAAAAACAAG |
| 12 | ko_phaC_B | CTTTTGTCCCAGCCGCTTCTGTTAACAAGCACAGCCCCGAC |
| 13 | ko_phaC_C | GTCGGGGCTGTGCTTGTTAACAGAAGCGGCTGGGACAAAAG |
| 14 | ko_phaC_D | gcatGAATTCCCTAAAACAATGGCGCCAACTAGTTC |
| 15 | phaP_A | atgcACGCGTttattcaaggaggaatttgattatgtcaac |
| 16 | phaP_B | gcatgagctcttattttacaactgcatattgctcaagag |
| 17 | phaOP_A1 | atgcCTCGAGAAAAAATTCAAAAATAAACAAAGATTTAGAATTG |
| 18 | phaC_B | gcatACGCGTTTATTTAGAGCGTTTTTCTAGCCAATCGCCG |
| 19 | phaP_for_PH | TATCATGTACAATGTCAACAGTAAAGTATGATACAG |
| 20 | phaP_rev_PH | ACAGCTCCTCGCCCTTGCTCACCATTTTTACAACTGCATATTGCT |
| 21 | GFP_for_PH | TCTTGAGCAATATGCAGTTGTAAAAATGGTGAGCAAGGGCGAGGA |
| 22 | GFP_rev_PH | TATCAGCGGCCGCTTACTTATACAGCTCGTCCATGCCGAGAGT |

Table S2 List of *B. megaterium* strains.

| **Strain** | **Genotype** | **Reference** |
| --- | --- | --- |
| DSM319 | Wildtype | DSMZ* |
| MS941 | Mutant of DSM319,  Δ*nprM* | [1] |
| GHH1 | Mutant of MS941,  Δ*upp* | This study |
| GHH3 | Mutant of GHH1, Δ*phaC* | This study |

*DSMZ - German Collection of Microorganisms and Cell Cultures

**Table S3 List of plasmids used in this study.**

| **Plasmid** | **Description** | **Reference** |
| --- | --- | --- |
| pMGBm19 | *B. megaterium* expression vector, chloramphenicol resistance | [2] |
| pUCTV2 | *B. megaterium* knock-out vector, temperature sensitive origin of replication, tetracycline resistance | [1] |
| PSMF2.1 | *B. megaterium* expression vector, tetracycline resistance | [3] |
| pEGFP-C1 | Mammalian expression vector for eGFP, kanamycin resistance | Clontech |
| pSMF2.1_SCCAA | *B. megaterium* expression vector, tetracycline resistance, containing *cyp11A1*, *adr* and *adx* | This study |
| pSMF2.1_CYP11A1eGFP | *B. megaterium* expression vector, tetracycline resistance, containing *cyp11A1egfp* fusion gene | This study |
| pSMF2.1_AdxeGFP | *B. megaterium* expression vector, tetracycline resistance, containing *adxegfp* fusion gene | This study |
| pSMF2.1_AdReGFP | *B. megaterium* expression vector, tetracycline resistance, containing adregfp fusion gene | This study |
| pUCTV2_Δ*upp* | *B. megaterium* knock-out vector, tetracycline resistance, containing flanking regions of the *upp* gene | This study |
| pUCTV2_Upp_Δ*phac* | *B. megaterium* knock-out vector, tetracycline resistance, containing the *upp* gene with its natural promoter and flanking regions of the *phaC* gene | This study |
| pSMF3_RBCP | *B. megaterium* expression vector, chloramphenicol resistance, containing *phaR*, *phaB*, *phaC* and *phaP* | This study |
| pSMF2.1_phaPeGFP | *B. megaterium* expression vector, tetracycline resistance, containing *phaP* | This study |

**Supplemental References**

1. Wittchen KD, Meinhardt F. Inactivation of the major extracellular protease from Bacillus megaterium DSM319 by gene replacement. Applied microbiology and biotechnology. 1995;42(6):871-7.

2. Gamer M, Frode D, Biedendieck R, Stammen S, Jahn D. A T7 RNA polymerase-dependent gene expression system for Bacillus megaterium. Applied microbiology and biotechnology. 2009;82(6):1195-203.

3. Bleif S, Hannemann F, Zapp J, Hartmann D, Jauch J, Bernhardt R. A new Bacillus megaterium whole-cell catalyst for the hydroxylation of the pentacyclic triterpene 11-keto-beta-boswellic acid (KBA) based on a recombinant cytochrome P450 system. Applied microbiology and biotechnology. 2012;93(3):1135-46.
